# Supplementary material for: Patterns of Intron Gain and Loss in Fungi
Source: PLoS Biol. 2004 Nov 30;2(12):e422. doi: 10.1371/journal.pbio.0020422 (PMC532390; doi:10.1371/journal.pbio.0020422)
Supplement: Table S1 — Also available at http://genes.mit.edu/NielsenEtAl/. (4.3 MB ZIP). [file pbio.0020422.st001.zip › NielsenEtAl/html/452.html]

AN1364.1.NCU06751.1.MG08563.1.FG01540.1


```
 CLUSTAL W (1.82) Multiple Sequence Alignments - Introns Inserted


Sequence 1: MG08563.1	228 aa
Sequence 2: FG01540.1	225 aa
Sequence 3: NCU06751.1	223 aa
Sequence 4: AN1364.1	227 aa
Alignment Length: 232 aa
Number Identitical Residues: 84 aa
Alignment Score (without introns) 4819


MG08563.1 	MS-HCHDEHGGDDHHHHHGEDGHDHSDDLTPALQNSLYTRIEFDKIVTLNE~DAHNSGLA
NCU06751.1	MSHHCHDEHDDH-HHHHEGGAEHDHSDDITPAVQFSLYQHINFDEITTLNE~QVHGSGQA
FG01540.1 	MS-HCHDEHTGHGHGHDHHE--HDHSDDITPAVQFSLYSQINFDHIVTLNE~ADRDAGLQ
AN1364.1  	MSHHHHSHNNGHGHCHGDGGDGHDHSNDITPALQSLLYSQIRFDSITTLNE1ATPQSGAA
          	** * *..: ...* * .    ****:*:***:*  ** :*.** *.****     :*  

MG08563.1 	VVKKTWAERLTPTPELASDADEQLLMHVP~FTGQVKLHSILVRTSDSDSAPRTLRVFANR
NCU06751.1	IIKKTWAERLATEPEVVSDADEQLIINVP2FTAQIKLHSVLLRTSPSPSAPRTLRLLANA
FG01540.1 	IVKKNWQERLSTEPELASDVDEQLLMTVP2FTAQIKLHSILIRTSPSASAPKTLHLFINR
AN1364.1  	IVKKTWAERQNDTPELESDADEQLLMYIP2FTGQVNVHSILIYTAPTPSAPRTLKLFKNR
          	::**.* **    **: **.****:: :* **.*:::**:*: *: : ***:**::: * 

MG08563.1 	PESFDFSAAESETPLQEIELSR---TSAVQELNVKRAKFGGVARLSLFFVDNFGDGDEDV
NCU06751.1	-DIHDFGQAEDSTPTQEFELSQ---TSEIQELPVKRAKFNAVQRLCLFFPDNFSQGEEDE
FG01540.1 	-DDLDFAAAEESDPVQTLELSQ---TGDLQEIPVKRALFGKVQRLVLFFADNFGDGDEDV
AN1364.1  	-DDLDFSTASELKPTQTIEVPQPIPGTDVFELPLNRAHWNATTSVTLFFEDNWSRGEEEV
          	 :  **. *..  * * :*:.:. .   : *: ::** :. .  : *** **:. *:*: 

MG08563.1 	TRLSYLGFKGEWMPLGRAPANILYEAAANPSDHALKGTSVNAMGSGIGGRGPGA
NCU06751.1	TRISYIGFKGEWMTLGQAPKNIVYEAAARPTDHKVKGT-ADARGVGYNRQG---
FG01540.1 	TRISYIAFKGEWTQLGRAPANIIYEAAANPGDHKLKGTDVNQMGSGIGGRGPGI
AN1364.1  	TKVGYIGLKGQFMALNREPVSFLYEAAANPNDHVAIPG-VNGVGGRIMPGQ---
          	*::.*:.:**::  *.: * .::*****.* **      .:  *
```
